# Birds select for higher mimetic accuracy, but imperfect mimics benefit from chromatic resemblance to their models

**DOI:** 10.1093/cz/zoaf071

**Published:** 2025-11-06

**Authors:** Anita Szabó, Barbora Váňová, Stano Pekár, Alice Exnerová

**Affiliations:** Faculty of Science, Department of Zoology, Charles University, Viničná 7, Prague 12844, Czech Republic; Faculty of Science, Department of Zoology, Charles University, Viničná 7, Prague 12844, Czech Republic; Faculty of Science, Department of Botany and Zoology, Masaryk University, Kotlářská 2, Brno 61137, Czech Republic; Faculty of Science, Department of Zoology, Charles University, Viničná 7, Prague 12844, Czech Republic

**Keywords:** ant mimicry, avian predators, batesian mimicry, imperfect mimicry, mimetic accuracy

## Abstract

Spiders have a high incidence of ant mimicry, exhibiting morphological, chromatic, and behavioral mimetic traits, but their mimetic accuracy varies greatly across taxa. Passerine birds may potentially select for accurate mimicry, but studies focused on their responses to ant mimics are surprisingly very scarce. In this study, we tested great tits (*Parus major*) as predators using photographs of ant-mimicking spiders, ants, and non-mimetic spiders to investigate the importance of morphological and chromatic traits for generalization from ant models to ant-mimicking spiders. Birds were divided into 2 groups, each trained to discriminate between non-mimetic spiders and either monochromatic (black) or dichromatic (black-reddish) ants, before being tested with mimics. Once birds from both groups learned to categorize correctly non-mimetic spiders and ants, they subsequently avoided morphologically accurate mimics, but only the birds trained to avoid dichromatic ants avoided morphologically inaccurate dichromatic mimics (e.g., *Micaria sociabilis*). Our results suggest that morphological and chromatic traits both play important roles in ant mimicry and chromatic traits can compensate for inaccurate morphological mimicry. Furthermore, classification of mimetic accuracy by birds corresponded roughly to the classification by humans.

## Graphical Abstract

**Graphical Abstract:**
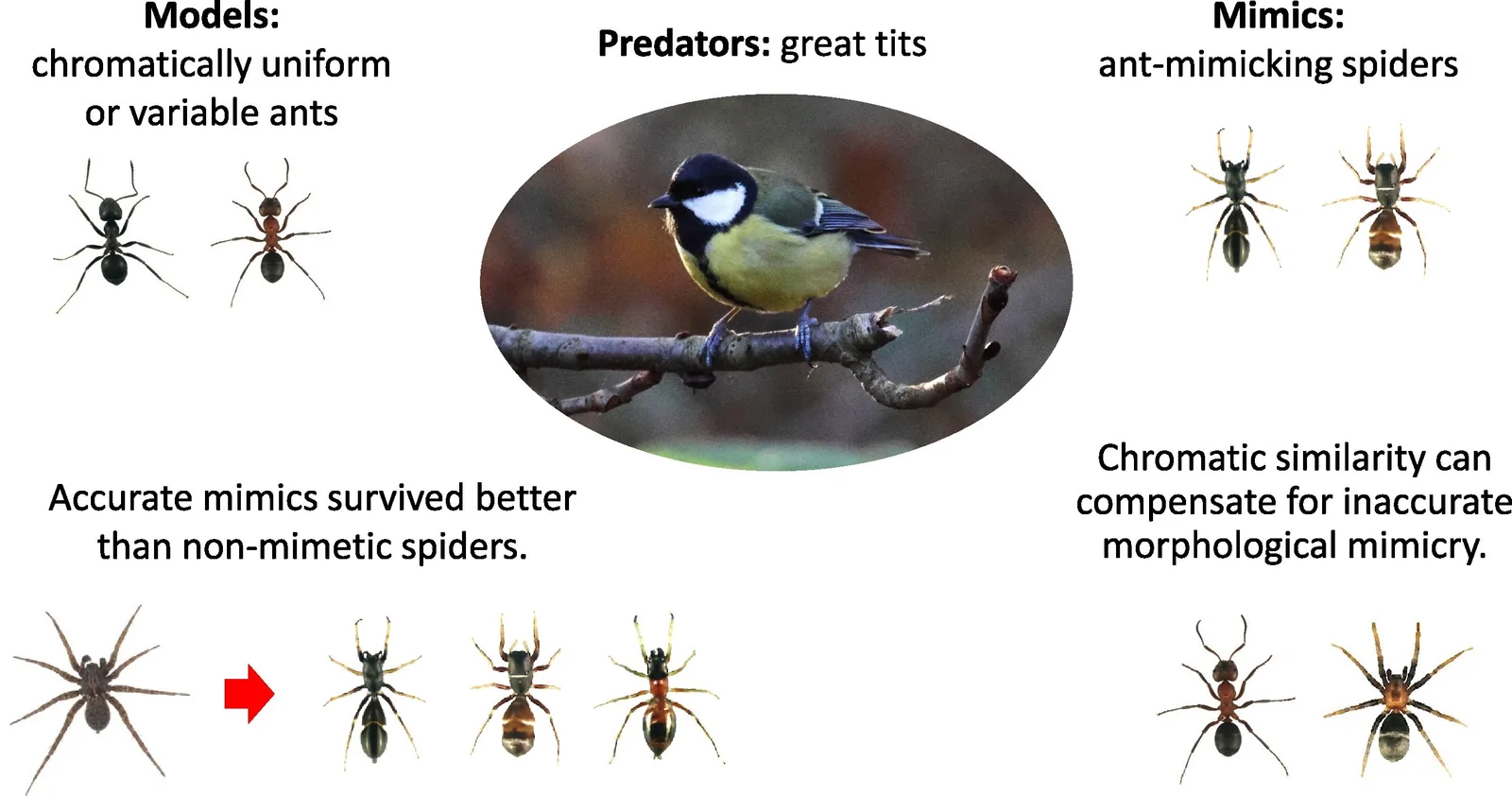

In Batesian mimicry, undefended mimics gain protection by resembling defended models (Bates 1862). This resemblance can involve several modalities, such as visual, acoustic, or olfactory (e.g., Dettner and Liepert 1994; Barber and Conner 2007). However, phenotypic resemblance to the model can vary among mimics, ranging from inaccurate to highly accurate mimicry (Kikuchi and Pfennig 2013; Sherratt and Peet-Paré 2017; Ruxton et al. 2018). The evolution of inaccurate mimicry could be the result of selective forces from different predators acting on mimics, a phenomenon termed “multiple-predator hypothesis” (Pekár et al. 2011). This hypothesis is supported by the finding that while some ant-mimicking spiders are protected from ant-avoiding predators of spiders, they could fall victim to predators specialized on attacking ants (Nelson et al. 2006b). Another possible reason could be that mimics are unable to increase their similarity to models due to morphological and genetic constraints (Kikuchi and Pfennig 2013). For instance, when mimics and models belong to phylogenetically distant taxa with different body plans as is the case of ant-mimicking spiders and ant models.

Inaccurate Batesian mimicry has been observed in butterflies, snakes, and hoverflies (Clarke and Sheppard 1960; Greene and McDiarmid 1981; Penney et al. 2014) and also in spiders which frequently mimic ants (e.g., Ceccarelli 2013; Pekár 2022). Ant-mimicking (myrmecomorphic) species can be found in at least 85 spider genera belonging to 16 families (Pekár 2014). Ants are suitable models as they are well defended against predators by several mechanisms including biting, stinging, and spraying formic acid and they also exhibit aggressive behavior and collective defense (Hölldobler and Wilson 1990). They are generally avoided by non-specialist predators (Nelson and Jackson 2006; Nelson et al. 2006a, 2006b; Venable et al. 2019). Most of the ant-mimicking species belong to salticid spiders (e.g., *Leptorchestes, Myrmarachne*), but they can also be found in corinnid (e.g., *Apochinomma, Castianeira*), thomisid (e.g., *Amyciaea, Aphantochilus*), gnaphosid (e.g., *Micaria*), and zodariid (e.g., *Storena, Zodarion*) spiders (Cushing 1997).

Besides resembling their ant models in color, shape, and size, myrmecomorphic spiders often exhibit locomotor mimicry (Pekár and Jarab 2011; Pekár et al. 2022), namely waving their forelegs imitating the antennal movement of ants, thus overcoming the constraint of having 4 pairs of legs and no antennae. Several studies found that even imperfect mimics can gain protection by moving fast and in a similar gait as ants, making it more difficult for visually-oriented predators to discriminate them from their models, thus compensating for their inaccurate visual resemblance (Pekár and Jarab 2011; Shamble et al. 2017; Zeng et al. 2023). Furthermore, Pekár et al. (2020) found that *Leptorchestes berolinensis* (C. L. Koch) undergoes transformational mimicry, namely juveniles are more similar to *Lasius* and *Colobopsis* ants, while adults resemble *Camponotus* or *Lasius* ants, with all stages exhibiting accurate mimicry.

Resembling ants can be beneficial in several ways. Ant-eating spiders gain protection by exhibiting a so-called aggressive mimicry, which allows them to capture their prey without being attacked by nest mates. Resembling ants can also protect mimics from visually-oriented predators, which is supported by the finding that myrmecomorphic spiders are most common among diurnal hunters, which actively search for prey (Pekár 2014). Several studies found that predators hunting for spiders such as jumping spiders (Salticidae) and mantises can be deceived into avoiding ant mimics (e.g., Nelson et al. 2006a, 2006b; Nelson 2012; Shamble et al. 2017). Furthermore, Nelson et al. (2006a, 2006b) found that 3 mantis species (*Loxomantis* sp., *Orthodera* sp., and *Statilia* sp.) showed innate aversion to ants and even naïve mantises generalized their avoidance to myrmecomorphic jumping spiders. Another study found that *Peckhamia picata* (Hentz), a mimetic jumping spider, exhibits not only visual mimicry, thus deceiving other predatory salticids (Durkee et al. 2011), but it also uses chemical cues to gain protection from its model ant (*Camponotus nearcticus* (Emery)) and from another chemically-oriented predator, the mud-dauber wasp *Sceliphron caementarium* (Drury) (Uma et al. 2013).

Birds represent another group of visually-oriented predators, which can play an important role in the evolution of visual mimicry in arthropods (Kazemi et al. 2018; Ruxton et al. 2018; Thurman and Seymoure 2016). Several studies have shown that birds primarily attend to color cues in prey discrimination learning (Aronsson and Gamberale-Stille 2008; Kazemi et al. 2014) and since myrmecomorphic spiders often resemble the color and/or the behavior of their models (Pekár et al. 2022), insectivorous birds represent a suitable predator model to evaluate the Batesian mimicry of ant-mimicking spiders. Moreover, ants are generally avoided by most avian species in the Western Palaearctic (except for a few specialists, such as woodpeckers; Cramp 1988; Cramp 1992; Cramp et al. 1993), whereas spiders constitute an important part of the diet of many birds (Gajdoš and Krištín 1997). Ant-mimicry may thus protect the otherwise undefended spiders from avian predation. So far, only 2 studies investigated the reaction of avian predators to myrmecomorphic spiders. Palmgren et al. (1937) tested 5 small passerine species and found that birds often preferred non-mimetic spiders or insects over *Myrmarachne formicaria* (De Geer). Furthermore, Veselý et al. (2021) showed that the mimic (*Phrurolithus festivus* (C. L. Koch)) gained protection from great tits (*Parus major* (Linnaeus)) even despite exhibiting imperfect mimicry.

In the present study, we used standardized photographs to assess the responses of great tits (*Parus major*) to several species of ant-mimicking spiders ranging from inaccurate to highly accurate mimics. The aim of the experiment was to investigate how ant model chromatic diversity affects prey categorization by avian predators and their generalization to differently accurate spider mimics. First, the birds completed a category discrimination training with ants and non-mimetic spiders and then they were presented with ant-mimicking spiders differing in their morphological and chromatic resemblance to ants. We hypothesized that the birds experienced with chromatic diversity of ant models would avoid a broader range of ant-mimicking spiders compared to birds experienced with uniformly colored ant models, as prey phenotypic diversity may lead to predators forming broad prey categories (Kikuchi et al. 2019) and facilitate the spread of imperfect mimics (Beatty et al. 2004). Alternatively, chromatic mimicry might compensate for inaccurate morphological mimicry of some species, since color appears to be the most salient discriminative cue for avian predators (Kazemi et al. 2014).

## Materials and methods

### Birds

We tested 36 wild-caught great tits *(Parus major*) during the years 2021–2023. The birds were caught using mist-nets in the Botanical garden of Charles University in Prague (50.08 N, 14.24 E, Czech Republic) from September to February each year. Upon capture, the birds were sexed, aged (yearlings vs. older adults; Svensson 1992) and assigned to one of the 2 treatment groups. Considering the STRANGE framework (Webster and Rutz 2020), we balanced the sex and age ratios between the experimental groups to avoid potential sampling biases. Birds were housed individually in indoor cages (50 × 40 × 50 cm), allowing a visual and acoustic contact with other birds in the aviary. Before the start of the experiment, the birds were habituated to the aviary environment for 2–5 days. Light conditions corresponded to the natural photoperiod. Birds were offered a diet consisting of mealworms (larvae of *Tenebrio molitor*), sunflower seeds, and commercial food mixtures for insectivorous birds (Uni Patee and Insect Patee, Orlux).

### Artificial prey

**Figure 1:**
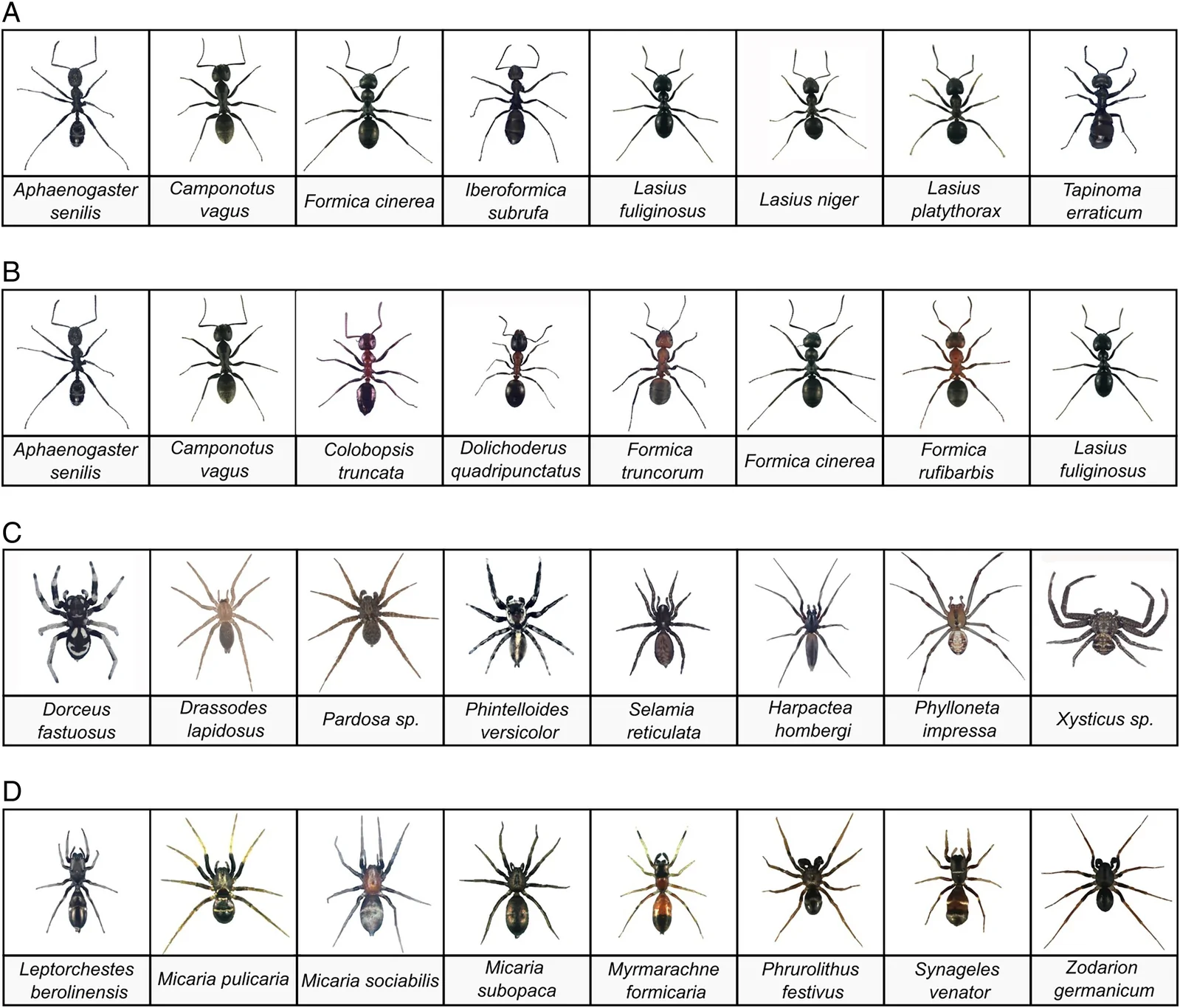
Set of photographs used in the discrimination training (A, B, C) and the generalization task (D). (A) Photographs of ant models used as unpalatable prey for the chromatically uniform treatment group. (B) Photographs of ant models used as unpalatable prey for the chromatically variable treatment group. (C) Photographs of non-mimetic spiders used as palatable prey for both treatment groups. (D) Photographs of ant-mimicking spiders used as palatable prey for both treatment groups in the generalization task.

As prey items, we used photographs of 29 arthropod species: 13 ant species (including 3 ant species used for both treatment groups), 8 mimetic spider species, and 8 non-mimetic spider species (Fig. 1, Supplementary Table S1). All species occur in the Western Palaearctic region, and the mimetic spider species represent various degrees of myrmecomorphy. The selection of study species was driven by their availability. The myrmecomorphic spiders are rare in the western Palearctic, and they all imitate either uniformly black or dichromatic black-reddish ant species. Beside *Dorceus fastuosus*, which was collected in the Negev desert, all species were collected in the Czech Republic. Only one of the species (*M. formicaria*) shows marked sexual dimorphism, thus only adult females were used. To pair mimics and putative models, we used information on their morphology, coloration, habitat, and co-occurrence in time from a previous study (Pekár 2021).

Spiders and ants were brought to the laboratory alive, then killed by exposing them to chloroform for a few minutes. Freshly killed specimens were mounted in a natural position (i.e., with stretched appendages) on cardboard. Specimens were illuminated from the lateral sides using 2 fluorescent bulbs (13W daylight ReptiGlo 2.0 UVB) with a similar light spectrum to natural light. Three images of every individual were taken at different focal depths from the dorsal view using an Olympus X12 stereomicroscope with an Olympus SC50 camera (resolution: 2,560 × 1,920 pixels). These images were then combined into one picture using Olympus Stream Motion software (version 1.9.4). Subsequently, the background was removed in Corel Photo-Paint X8. We used 2 sets of photographs of different individuals of each species to avoid providing additional visual cues (such as position of antennae and legs) to birds. The body length was standardized across ant and spider species. The pictures were placed on white background (GNU Image Manipulation Program) and printed on a white 350 g/m^2^ cardboard paper (Xerox Versant printer) using a color profile FOGRA 39 to maintain color accuracy.

Prey photographs were presented to the birds on cardboard paper squares (25 × 25 mm) baited with a piece of mealworm underneath. The pieces of mealworms were either injected with water (palatable prey) or with a 15% quinine-phosphate solution (unpalatable prey) before each trial. Quinine is an odorless substance with an intensively bitter taste and makes food aversive for birds (Lindström 1999; Ham et al. 2006). Birds were not allowed to see the pieces of mealworms, only the cards with the photograph of the prey. The prey items were presented to the birds one by one in glass Petri dishes against a white background. Using prey images and artificial prey items represents a suitable approach for testing prey discrimination and generalization in avian predators (e.g., Dittrich et al. 1993; Rönkä et al. 2018), where experiments with real prey would not be feasible for practical or ethical reasons.

### Experimental setup

Experiments were conducted in wooden cages (70 × 70 × 70 cm) with wire-mesh side walls and a front wall made of one-way glass through which the birds were observed. These cages contained a wooden perch, a water bowl and a rotating wooden tray with 6 circular cups. The cages were illuminated by Biolux Combi 18W (Osram) bulbs to simulate natural light conditions. Before each experiment, birds were food deprived for 2 hours to increase their foraging motivation (Ihalainen et al. 2008; Exnerová et al. 2015). Water was accessible ad libitum throughout the experiment.

### Experimental design

Prior to the experiment, birds were allowed to habituate to the experimental cage and pre-trained to search for pieces of mealworms placed underneath a white paper card (25 × 25 mm) in one of the cups in the feeding tray. The pre-training was considered successful when the birds learned to turn the card and ate the piece of mealworm stuck underneath 3 times in a row.

Then, the birds participated in categorization training, during which they learned to discriminate between 8 species of ants (unpalatable prey) and 8 species of non-mimetic spiders (palatable prey). For the categorization training, the birds were divided into 2 groups: 1) the uniform-models group was trained to avoid chromatically uniform (black) ant species and 2) the variable-models group was trained to avoid chromatically variable (black and black-reddish) ant species (Fig. 1). The categorization training consisted of daily blocks of 16 trials, each lasting for 2 minutes, with one prey item offered at a time. The sequence of presented prey species followed a constrained-randomization design in which no more than 2 consecutive palatable or unpalatable prey items were used in a row. During each trial, we recorded 1) whether the bird handled the prey (touching or turning the paper card with the beak), 2) latency to handling, and 3) whether the bird tasted the piece of mealworm. In case the bird did not taste and at least partly ate the palatable bait, we repeated the trial to make sure that the birds received the reward for handling the palatable prey item, however the result of the first decision (i.e., prey left untouched) was recorded into the protocol. Furthermore, during the first block of categorization training, birds had to taste at least 4 unpalatable prey items to get sufficient experience with prey unpalatability; otherwise, the trials were repeated. Leaving the unpalatable prey untouched and eating at least part of the palatable bait were considered correct decisions. To continue to the next phase of the experiment (generalization task), the birds had to reach the learning criterion of 85% correct decisions per block on 3 different days. Four birds (all trained with uniformly black ants), which did not reach this criterion, were excluded from further testing.

Once the birds passed the discrimination-learning criterion, they participated in the generalization task, which consisted of 2 daily blocks of 24 trials. We used the same species of non-mimetic spiders and ants as during the categorization training with the addition of 8 species of ant-mimicking spiders (baited with palatable mealworms). We conducted the generalization task in 2 daily blocks to assess the effect of experience with palatable mimics on the first day on the attack rate of mimics and models on the second day. Each trial lasted for 2 minutes, and one prey item was offered at a time in a constrained-randomization design to ensure that birds were not offered more than 3 consecutive prey species of the same category in a row. In each trial, we recorded 1) whether the bird handled the prey item, 2) latency to handling, and 3) whether the bird ate (or attempted to eat) the pieces of mealworms. All trials were recorded using Observer XT 8.0^©^ Noldus and a video camera.

### Statistical analyses

To assess the speed of discrimination learning, we analyzed the number of training blocks the birds needed to reach the learning criterion by the Generalized Linear Model with quasi-Poisson error distribution and log link (GLM-qp) including the treatment group, age, and sex of the birds as predictors. To model the discrimination learning, we fitted a logistic curve for ants and non-mimetic spiders combined using the Generalized Linear Mixed Model with binomial error distribution and logit link (GLMM-b) from the mgcv package (Wood 2017). The fixed effects included the treatment group, age, and sex of the birds and the random effects included the intercept. We fitted several models with several random effects (only intercepts, only slopes, both) and selected the best model using AIC.

To assess the effect of experience on the birds' behavior toward mimics during the generalization task, we compared 1) numbers of mimics left untouched using GLM with Poisson error distribution and log link (GLM-p) that included the treatment group, sex, and age of the birds as predictors and 2) the attack probability on each ant-mimicking spider using Generalized Estimating Equation with binomial error distribution and logit link (GEE-b) from the geepack package (Yan 2002). GEE is an extension of GLM for correlated data that fits marginal model (Pekár and Brabec 2018). The model included the treatment group, the mimic species, and the group:species interaction as fixed effects. Post-hoc comparisons between treatment groups were done using pairwise Tukey tests from the emmeans package (Lenth 2025). To compare the attack probability on mimics within treatment groups, we used GEE-b separately for each group. Post-hoc comparisons were based on contrasts using a non-mimetic spider (*Pardosa* sp.) as a reference level. We performed the same analyses for the first and second day of the generalization task. The latencies to attack individual mimics were analyzed by Cox Proportional-Hazards model (Cox PHM) from the coxme package (Therneau 2024), which included the treatment group, the mimic species and the day of the generalization task as fixed effects and the bird identity as the random effect. We also compared the numbers of incorrect decisions (either attacking ants or leaving the non-mimetic spiders untouched) made by birds between the 2 days of the generalization task using GLM-p.

We used Spearman correlation to compare the assessment of mimetic accuracy of the mimics between humans and birds, separately for the groups trained with uniform and variable ant models, as the relationship was not linear and both scores were estimated with an error. The assessment scores by human observers were taken from a former study (Pekár 2021), where the same images of mimics were used. The assessment scores by birds were calculated as the proportion of individuals leaving a given mimic species untouched.

All analyses were done in R version 4.0.0 (R Core Team 2020).

## Results

### Discrimination training

When comparing the speed of discrimination learning, we found no significant difference between the groups trained with uniform and variable ant models (GLM-qp: df = 1, χ^2^ = 0.1, *P* = 0.75), the 2 age categories (GLM-qp: df = 1, χ^2^ = 1.18, *P* = 0.28) or between males and females (GLM-qp: df = 1, χ^2^ = 0.2, *P* = 0.65). The average number of training blocks needed to reach the learning criterion was 12 (±2.88) for the uniform-models group and 11.69 (±2.6) for the variable-models group.

**Figure 2:**
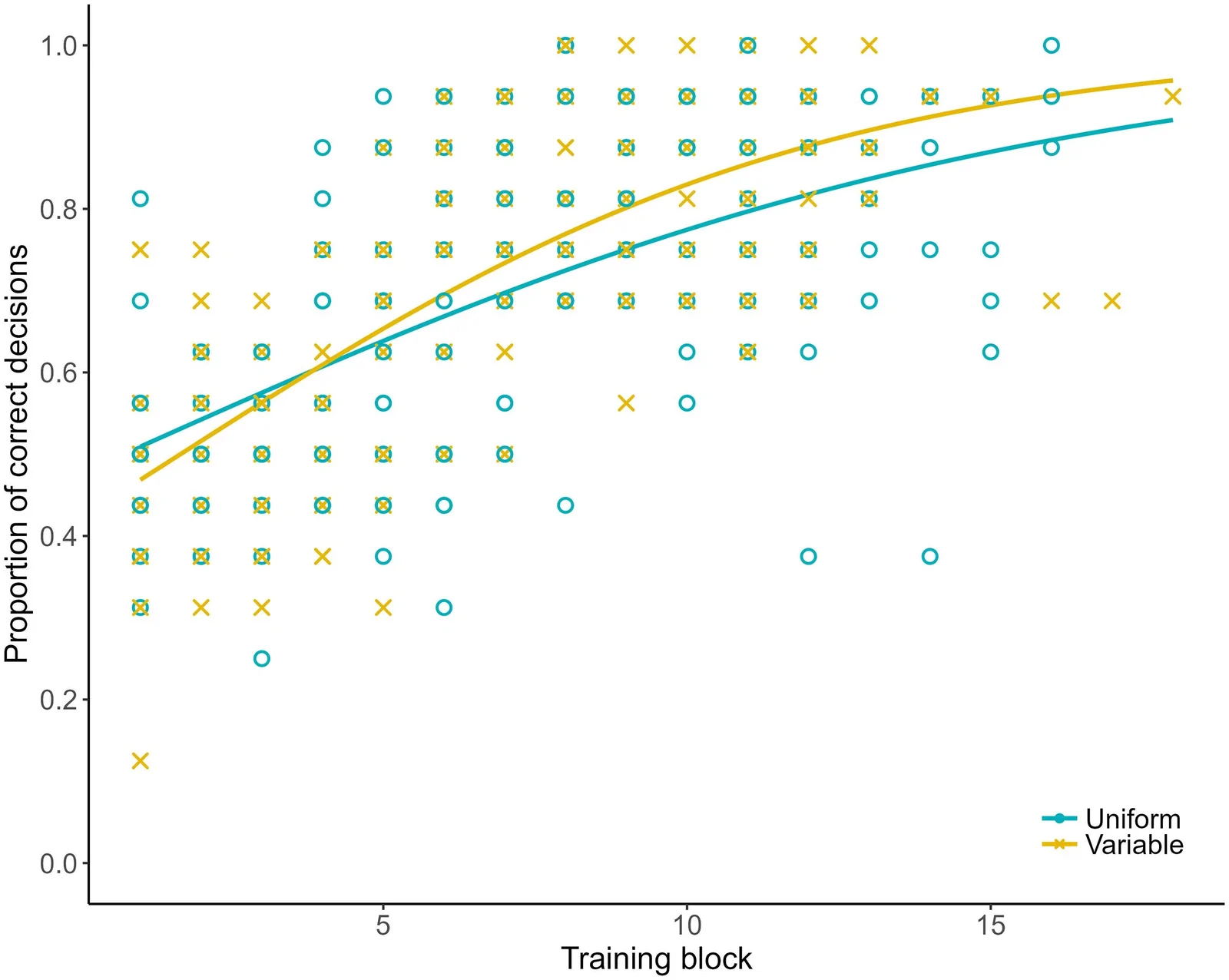
Relationship between training block number and the probability of correct decisions. Blue and yellow lines are estimated logit models for each group.

The probability of correct decisions (rejecting ants and accepting non-mimetic spiders) increased significantly with the number of training blocks (GLMM-b: df = 1, F = 217.1, *P* < 0.001, Fig. 2, Supplementary Figures S1 and S2). However, there was no significant difference between the groups trained with uniform and variable ant models (GLMM-b: df = 1, F = 3.4, *P* = 0.066), the 2 age categories (GLMM-b: df = 1, F = 2.6, *P* = 0.11) or between males and females (GLMM-b: df = 1, F < 0.1, *P* = 0.99). The effect of random slopes and intercepts was significant (GLMM-b: edf = 20.7, F = 77.8, *P* < 0.001). The level of 85% correct decisions was achieved after 10 training blocks.

### Generalization tasks

**Figure 3:**
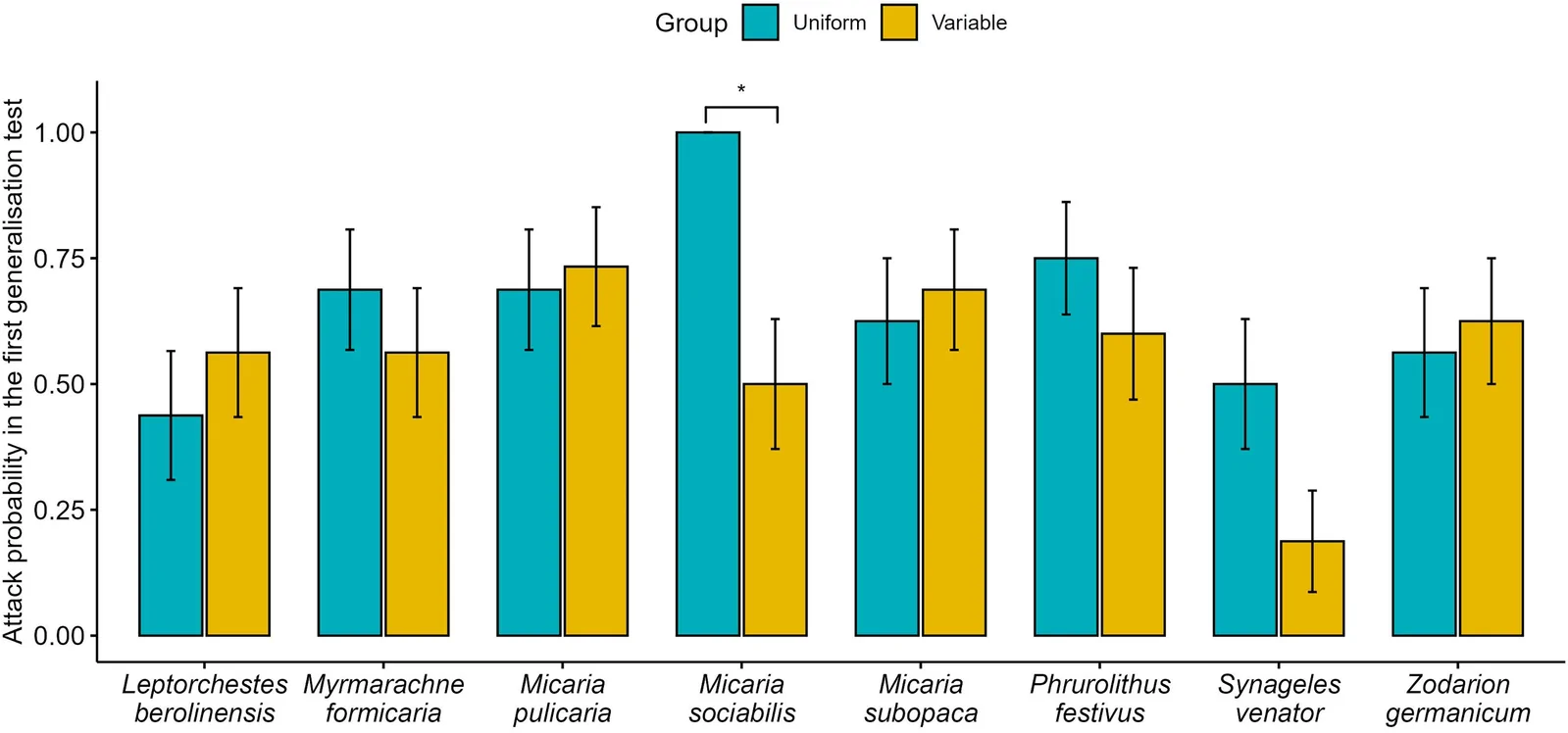
Attack probability on the different ant-mimicking spider species on the first day of the generalization task, compared between the 2 treatment groups. The asterisk denotes the significant difference based on pairwise post-hoc comparisons with Tukey test (* *P* = 0.02). Bars show means, vertical lines represent 95% confidence intervals.

We found that the number of mimics left untouched during the first day of generalization task was not different between the 2 treatment groups (GLM-p: df = 1, χ^2^ = 46.75, *P* = 0.23) and also did not depend on age (GLM-p: df = 1, χ^2^ = 46.3, *P* = 0.55) and sex of the birds (GLM-p: df = 1, χ^2^ = 46.71, *P* = 0.84). However, the attack probability differed among the ant-mimicking spider species (GEE-b: df = 7, χ^2^ = 16.98, *P* = 0.02), and although there was no main effect of the treatment group (GEE-b: df = 1, χ^2^ = 1.18, *P* = 0.28), the differences between mimics tended to depend on the group (GEE-b: df = 7, χ^2^ = 12.44, *P* = 0.09). When comparing the attack probability on individual mimics between the treatment groups, we found that *Micaria sociabilis* was attacked less often by the birds from the variable-models group compared to the uniform-models group (Tukey: t = 2.36, *P* = 0.02) and we also found a similar trend for *Synageles venator*, although not significant (Tukey: t = 1.81, *P* = 0.07; Fig. 3). The proportion of attacks for the remaining 6 mimic species were not significantly different between the treatment groups. Comparing the proportions of attacks on different mimic species within each treatment group, we found significant differences among species in both groups (GEE-b _Uniform_: df = 8, χ^2^ = 20.26, *P* < 0.001; GEE-b _Variable_: df = 8, χ^2^ = 30.7, *P* < 0.001). Birds from the uniform-models group attacked *Synageles venator* (contrast, *P* = 0.001), *Zodarion germanicum* (contrast, *P* = 0.01), *Micaria subopaca* (contrast, *P* = 0.03), *Myrmarachne formicaria* (contrast, *P* = 0.05) and *Leptorchestes berolinensis* (contrast, *P* = 0.02) significantly less often than the non-mimetic *Pardosa* sp. In the variable-models group, almost all the mimics (*Zodarion germanicum*: *P* = 0.03, *Synageles venator*: *P* < 0.001, *Phrurolithus festivus*: *P* = 0.02, *Micaria subopaca*: *P* = 0.05, *Micaria sociabilis*: *P* = 0.03, *Myrmarachne formicaria*: *P* = 0.05, *Leptorchestes berolinensis*: *P* = 0.05) were attacked less often compared to the non-mimetic *Pardosa* sp.

**Figure 4:**
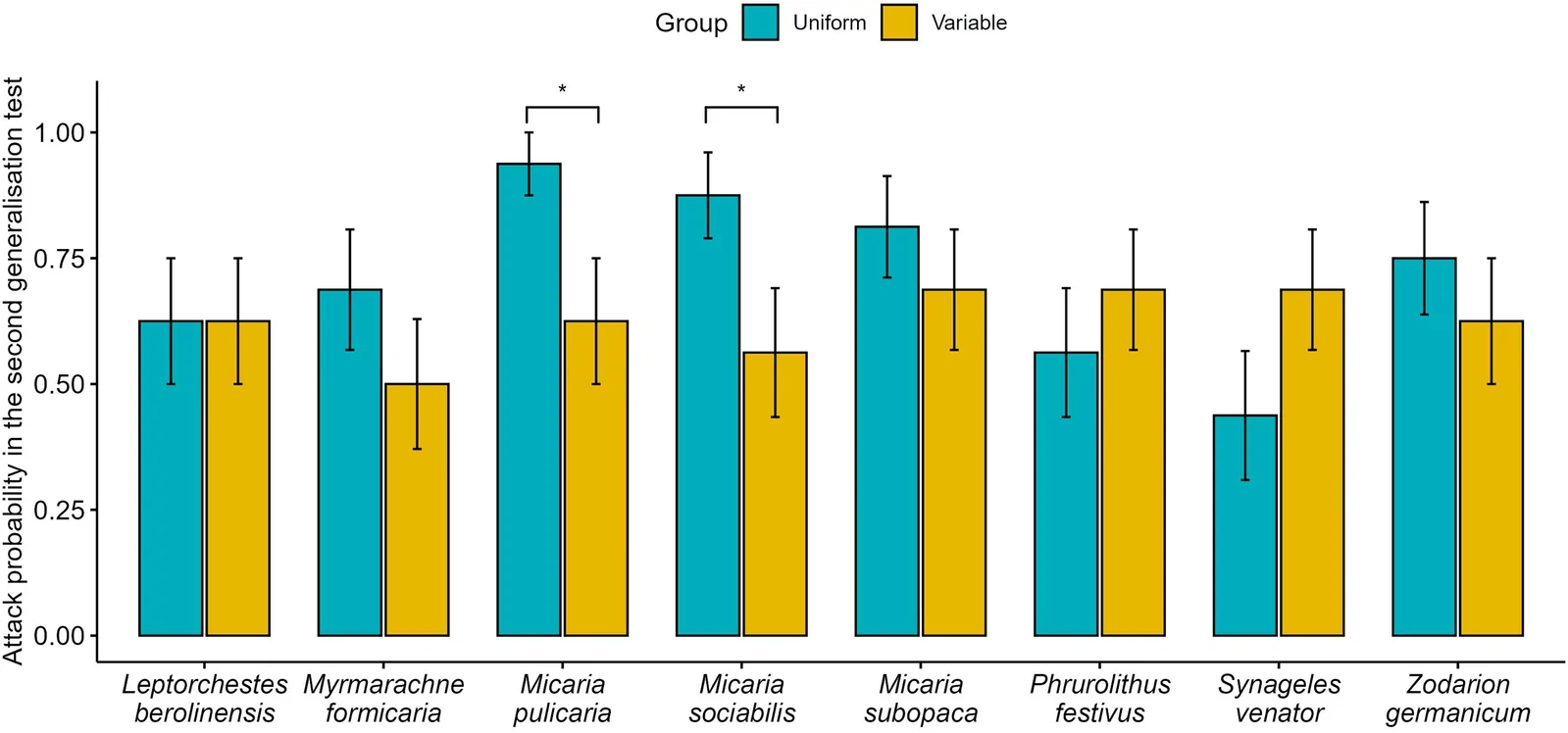
Attack probability on the different mimic species during the second day of generalization, compared between the 2 treatment groups. The asterisks denote significant difference based on pairwise post-hoc comparisons with Tukey test (* *P* = 0.06). Bars show means, vertical lines represent 95% confidence intervals.

When analyzing the performance of birds in the second day of the generalization task, we found that the number of mimics left untouched did not differ between the 2 treatment groups (GLM-p: df = 1, χ^2^ = 50.16, *P* = 0.23) and also did not depend on age (GLM-p: df = 1, χ^2^ = 49.95, *P* = 0.74) and sex of the birds (GLM-p: df = 1, χ^2^ = 50.1, *P* = 0.74). There was a significant interaction between mimic species and group in overall attack probability (GEE-b: df = 7, χ^2^ = 23.18, *P* = 0.002). When comparing the attack probability on individual mimics between the treatment groups, we found a tendency for birds from the variable-models group to attack *Micaria sociabilis* (Tukey: t = 1.87, *P* = 0.06) and *Micaria pulicaria* (Tukey: t = 1.9, *P* = 0.06) less often compared to the uniform-models group (Fig. 4). Comparing the proportions of attacks on different mimic species within each treatment group, we found significant differences among species in both groups (GEE-b_Uniform_: df = 8, χ^2^ = 94.4, *P* < 0.001; GEE-b_Variable_: df = 8, χ^2^ = 25.2, *P* = 0.001). Birds from the uniform-models group attacked *Synageles venator* (contrast, *P* = 0.005) and *Phrurolithus festivus* (contrast, *P* = 0.05) less often than the non-mimetic *Pardosa* sp. In the variable-models group, *Micaria sociabilis* (contrast, *P* = 0.05), *Micaria pulicaria* (contrast, *P* = 0.03) and *Myrmarachne formicaria* (contrast, *P* = 0.008) were attacked less frequently compared to the non-mimetic *Pardosa* sp.

**Figure 5:**
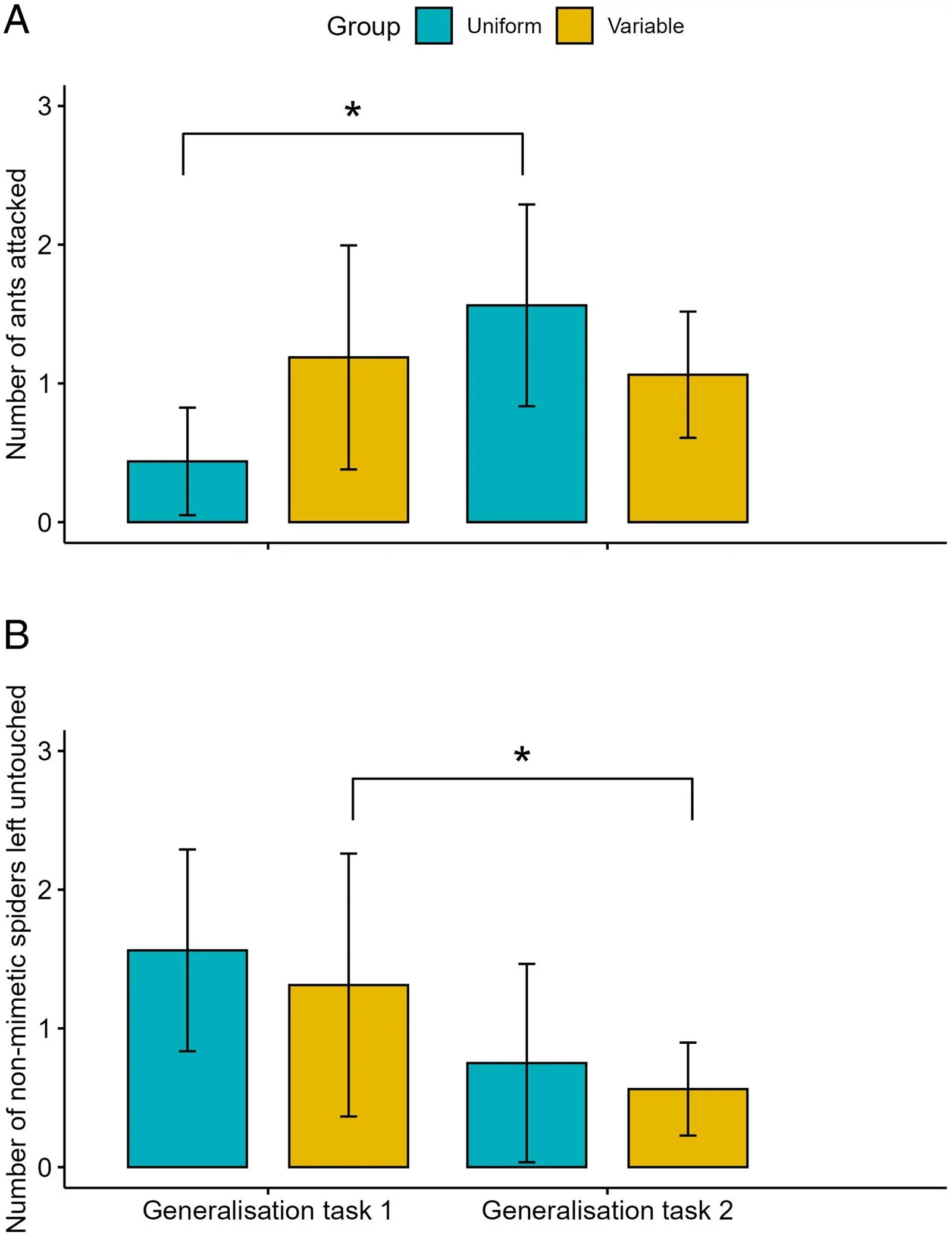
The number of incorrect decisions compared between the 2 days of the generalization task and 2 treatment groups. (A) The number of ant models attacked. The asterisk denotes the significant difference based on GLM-p (* *P* = 0.05). Bars show means, vertical lines represent 95% confidence intervals. (B) The number of non-mimetic spiders left untouched. The asterisk denotes the significant difference based on GLM-p (*P < 0.05). Bars show means, vertical lines represent 95% confidence intervals.

When comparing the performance of birds between the 2 days of the generalization task, we found significant differences in the number of incorrect decisions: birds from the uniform-models group tended to make more mistakes toward attacking ants (GLM-p: df = 1, χ^2^ = 3.8, *P* = 0.05; Fig. 5A) and birds from the variable-models group made less mistakes leaving non-mimetic spiders untouched during the second day of the generalization task (GLM-p: df = 1, χ^2^ = 9.56, *P* = 0.002; Fig. 5B).

**Figure 6:**
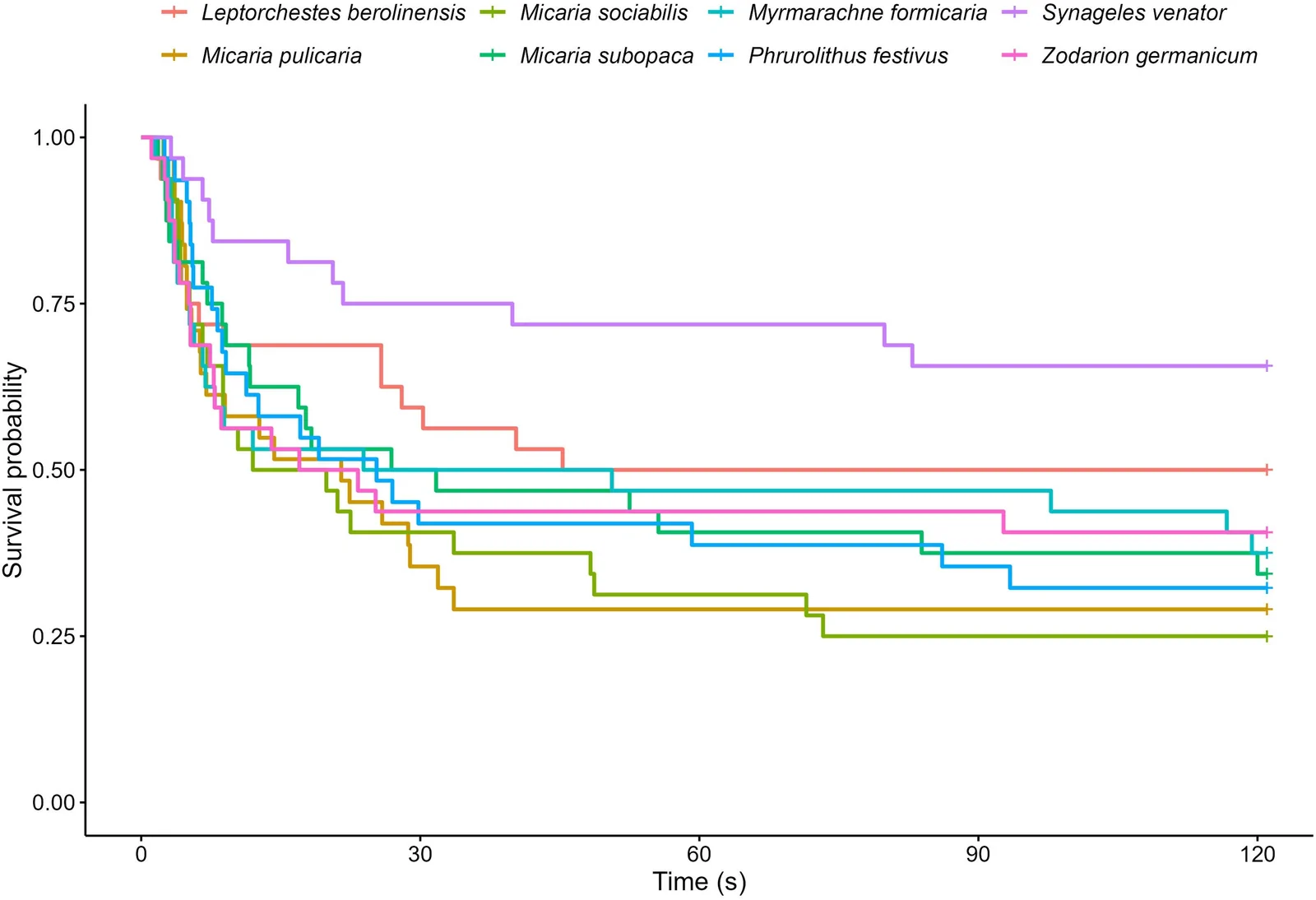
Survival probability change in time for each mimic species during the first day of the generalization task.

The latencies to attack mimics were significantly different among species (Cox PHM, χ^2^ = 24.3, *P* = 0.001; Fig. 6). Compared to the shortest attack latencies for *Micaria pulicaria,* the latencies were significantly longer for *Leptorchestes berolinensis* (contrast, *P* = 0.03), *Myrmarachne formicaria* (contrast, *P* = 0.04), *Phrurolithus festivus* (contrast, *P* = 0.04) and *Synageles venator* (contrast, *P* < 0.001). There was no effect of treatment group (Cox PHM: χ^2^ = 0.52, *P* = 0.47) or the day of the generalization task (Cox PHM: χ^2^ = 1.06, *P* = 0.3).

### Avian vs human assessment

**Figure 7:**
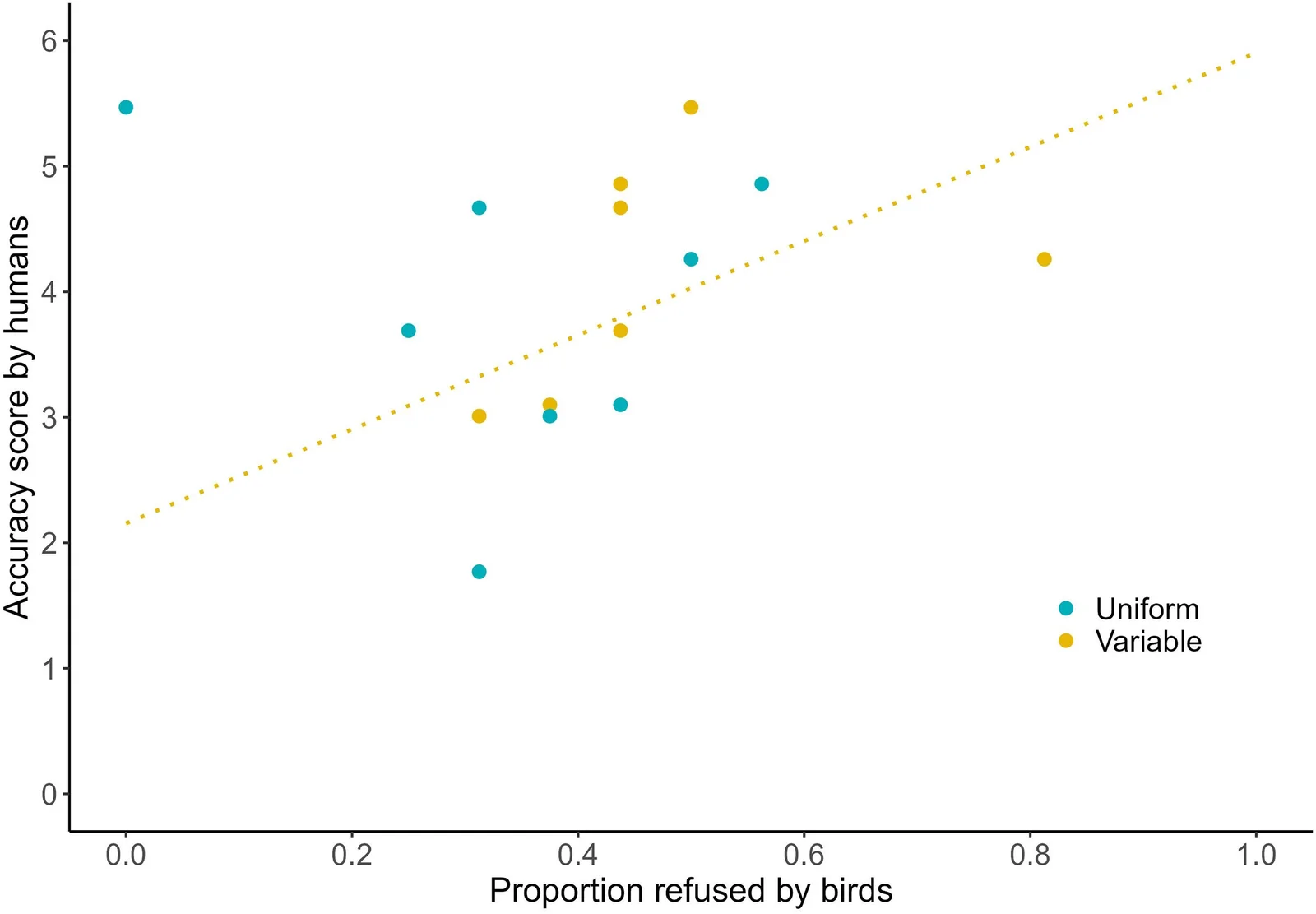
Relationship between the accuracy scores of ant-mimicking spider species by humans and the proportions refused by birds experienced with uniform and variable ant models. Dashed line marks significant correlation.

For the bird group trained with uniform ants we did not find a significant correlation (Spearman's rank correlation, rho = −0.06, S = 89.03, *P* = 0.888), whereas for the group trained with variable ants we found a significant positive correlation (Spearman's rank correlation, rho = 0.80, S = 16.98, *P* = 0.017) with the human assessment of mimetic accuracy of myrmecomorphic spiders (Fig. 7).

## Discussion

Birds in our experiment learned to categorise ants and non-mimetic spiders irrespective of the treatment group. This result indicates that even though birds primarily tend to rely on chromatic cues in prey discrimination learning (Kazemi et al. 2014), the chromatic diversity among ant models did not make the task considerably more difficult compared to the uniform-models treatment.

Comparing the performance of the 2 treatment groups on the first day of the generalization task, we did not find support for the hypothesis that the experience with higher chromatic diversity of ant models during the category discrimination learning could lead to broader generalization to differently accurate mimics. However, we found that one of the ant-mimicking spiders (*Micaria sociabilis*) was attacked by most birds in the uniform-models group while birds in the variable-models group tended to avoid this species. This is an interesting finding since *M. sociabilis* is not considered an accurate mimic of ants by human observers (Pekár 2021), thus we did not expect any of the groups to avoid it. A possible explanation for this result could be that *M. sociabilis* possesses reddish coloration, which could have deceived birds from the variable-models group as they learned to avoid both black and black-reddish ants. This finding supports the hypotheses that birds primarily attend to color when deciding whether to attack prey (e.g., Exnerová et al. 2006; Aronsson and Gamberale-Stille 2008; Kazemi et al. 2018). Another mimetic spider, which was attacked slightly less often by the birds in the variable-models group was *Synageles venator*, which also has reddish color markings on the dorsal side of the opisthosoma. Although this species possesses a generally shiny black coloration and elongated body similar to ants, human observers rated it as a rather imperfect mimic (Pekár 2021). In contrast, another mimic species possessing a reddish coloration, *Myrmarachne formicaria*, was avoided equally by the birds from both treatment groups, possibly because it has accurate morphological mimicry. Analyzing the performance of birds during the second day of the generalization task revealed that *M. sociabilis* was still avoided by the variable-models group slightly more than by the uniform-models group and interestingly, the attack rate on another mimic (*Micaria pulicaria*) also tended to differ between groups as more birds from the variable-models group avoided this species. Although all 3 species avoided by birds from the variable-models group were rated as imperfect mimics by human observers (Pekár 2021), it seems that for avian predators, resemblance in coloration can compensate for inaccurate morphological mimicry.

We found that the attack latencies differed depending on the mimic species. *Micaria pulicaria* was attacked at the shortest latencies, while *Synageles venator*, *Leptorchestes berolinensis*, *Myrmarachne formicaria* and *Phrurolithus festivus* took longer to attack. This could indicate that *M. pulicaria* was perceived as a rather inaccurate mimic by the birds and they quickly decided to attack it, while the 4 other species exhibited a more convincing resemblance to ants, thus birds hesitated longer before attacking. Supporting this hypothesis, we found that *S. venator. L. berolinensis*, and *M. formicaria* survived better than the non-mimetic *Pardosa* sp. in both treatment groups during the first day of the generalization task. In the natural encounter, longer predator hesitation could convey better protection also by helping the prey to escape (Sherratt et al. 2023).

Although, the “eye of the beholder” hypothesis states that to assess mimetic accuracy, we have to take into account the visual system of the potential predator (Cuthill and Bennett 1993), human observers are sometimes used to assess mimetic accuracy both in Batesian (Pekár 2021) and Müllerian mimics (Beatty et al. 2004) and in case of hoverflies (*Syrphidae*), ratings of mimetic accuracy by humans were similar to those of pigeons (Dittrich et al. 1993; Golding et al. 2005; Penney et al. 2012). Our results show that humans assessed the accuracy of myrmecomorphic spiders similarly to birds. Specifically, the mimic species rated least accurate by humans (*M. pulicaria*) was also the one, which was attacked with the shortest latencies by birds. However, while the birds in our experiment rated *S. venator*, *L. berolinensis*, and *Z. germanicum* as accurate mimics, human observers did not consider these species highly accurate mimics (Pekár 2021). One reason for these differences could be that human observers were presented with a wider variety of ant mimics (including highly accurate Australian species), thus it is possible that the European mimics, which our birds were presented with, did not seem so accurate. Furthermore, humans were presented the mimics together with their models, whereas birds were presented each species separately. On the other hand, our results could indicate that birds might pay attention to other features than humans when assessing mimetic accuracy, such as color patterns and antennal length (Bain et al. 2007). Thus, the differences between humans and avian predators in the assessment of mimetic accuracy of some myrmecomorphic spiders could be either a result of partly different methods or it could be caused by different cues used for discriminating between similarly looking prey.

We found that birds were more likely to attack ants and less likely to leave the non-mimetic spiders untouched during the second day of the generalization task compared to the first day. This indicates that the experience with palatable mimics during the first day of the generalization task had an effect on birds' decisions on the second day. However, the effect depended on the treatment group as the 2 groups continued to be presented chromatically uniform or variable ant models during the generalization tasks. Specifically, the birds from the uniform-models group were more prone to attack ant models previously learned to be unpalatable, and birds from the variable-models group were less cautious to accept non-mimetic spiders as palatable prey. These results support the hypothesis that the presence of Batesian mimics may be harmful to their models as repeated encounters with palatable mimics can make the predators more inclined to sample the defended models, thereby increasing their predation risk (Lindström et al. 1997; Ruxton et al. 2018).

Another important strategy of ant-mimicking spiders is locomotor mimicry, which is exhibited across several species from the genus *Myrmarachne* (Nelson and Card 2016). Furthermore, Pekár (2021) showed that myrmecomorphic spiders became more difficult to identify for humans with increasing speed of movement, thus fast movement could be another possibility for mimics with inaccurate visual resemblance to escape predation. Therefore, it is possible that since we only used static images of ant-mimicking spiders, birds had more time to notice potential differences between the mimics and their models, and this could be the reason why we did not find more differences in the overall survival rates among mimetic spider species.

While using a single avian species may limit the generalization of our results, we believe that great tits represent a suitable model of natural avian predators of ant-mimicking spiders, since they are generalist predators of arthropods, and their diet includes a high proportion of spiders, especially in the breeding season (Cramp et al. 1993; Gajdoš and Krištín 1997). Their visual system (Ödeen et al. 2011) and behavior toward defended prey (Exnerová et al. 2003) are also representative of other small passerines, such as warblers and chats, which are potential predators of ant-mimicking spiders. So far, only one study tested the reactions of great tits to live individuals of a myrmecomorphic spider species (*Phrurolithus festivus*) and found that birds can be deceived even by imperfect mimics (Veselý et al. 2021). Furthermore, Palmgren et al. (1937) tested European robins (*Erithacus rubecula*), common redstarts (*Phoenicurus phoenicurus*), garden warblers (*Sylvia borin*), common whitethroats (*Sylvia communis*) and European pied flycatchers (*Ficedula hypoleuca*) and although the sample size was small, he found evidence that small passerines avoid *Myrmarachne formicaria* (De Geer) when presented together with non-mimetic spiders or insects.

In conclusion, we found that birds successfully learned to discriminate between ants and non-mimetic spiders irrespective of the degree of chromatic variation among ant species. The birds experienced with chromatic variation in the ant category during the discrimination training generalised their experience to some of the morphologically inaccurate mimics that resembled the coloration of ant models. Color may therefore represent an important component of the mimetic resemblance in ant-mimicking spiders and chromatic similarity can at least partly compensate for inaccurate morphological mimicry even in the absence of prey movement.

## Supplementary Material

zoaf071_Supplementary_Data
