# Supplementary material for: Birds select for higher mimetic accuracy, but imperfect mimics benefit from chromatic resemblance to their models
Source: Curr Zool. 2025 Nov 6;72(4):568–78. doi: 10.1093/cz/zoaf071 (PMC13500164; doi:10.1093/cz/zoaf071)
Supplement: Supplementary file 1 [file zoaf071_supplementary_data.docx]

**Supplementary material**

Table S1. List of species used in the experiment. Family classifications are as follows: Dysderidae: *Harpactea*; Eresidae: *Dorceus*; Formicidae: *Aphaenogaster*, *Camponotus*, *Colobopsis*, *Dolichoderus*, *Formica*, *Iberoformica*, *Lasius*, *Tapinoma*; Gnaphosidae: *Drassodes*, *Micaria*; Lycosidae: *Pardosa*; Phrurolithidae: *Phrurolithus*; Salticidae: *Leptorchertes*, *Myrmarachne*, *Phintelloides*, *Synageles*; Theridiidae: *Phylloneta*; Thomisidae: *Xysticus*; Zodariidae: *Selamia*, *Zodarion*.

| **Ant models** | **Non-mimetic spiders** | **Myrmecomorphic spiders** |
| --- | --- | --- |
| *Lasius niger* (Linnaeus) | *Dorceus fastuosus* (Koch) | *Micaria sociabilis* (Kulczyński) |
| *Aphaenogaster senilis* (Mayr) | *Drassodes lapidosus (*Walckenaer*)* | *Micaria subopaca* (Westring) |
| *Camponotus vagus* (Scopoli) | *Pardosa* sp. | *Micaria pulicaria* (Sundevall) |
| *Iberoformica subrufa* (Roger) | *Phintelloides versicolor* (Koch) | *Phrurolithus festivus* (Koch) |
| *Formica cinerea* (Mayr) | *Selamia reticulata* (Simon) | *Zodarion germanicum* (Koch) |
| *Lasius fuliginosus* (Latreille) | *Harpactea hombergi* (Scopoli) | *Synageles venator* (Lucas) |
| *Lasius platythorax* (Seifert) | *Phylloneta impressa* (Koch) | *Leptorchertes berolinensis* (Koch) |
| *Tapinoma erraticum* (Latreille) | *Xysticus* sp. | *Myrmarachne formicaria* (De Geer) |
| *Colobopsis truncata* (Spinola) |  |  |
| *Dolichoderus quadripunctatus*  (Linnaeus) |  |  |
| *Formica truncorum* (Fabricius) |  |  |
| *Formica rufibarbis* (Fabricius) |  |  |
| *Formica subrufa (*Roger*)* |  |  |

**Fig. S1 caption:** Relationship between training block number and the probability of rejecting ants. Blue and yellow lines are estimated logit models for each group.

**Alt text:** Scatter-plot showing the relationship between training block number and the probability of rejecting ants. Lines represent estimated logit models for each group.

**Fig. S2 caption:** Relationship between training block number and the probability of accepting non-mimetic spiders. Blue and yellow lines are estimated logit models for each group.

**Alt text:** Scatter-plot showing the relationship between training block number and the probability of accepting non-mimetic spiders. Lines represent estimated logit models for each group.
